# Mitochondrial metabolic determinants of multiple myeloma growth, survival, and therapy efficacy

**DOI:** 10.3389/fonc.2022.1000106

**Published:** 2022-09-16

**Authors:** Remya Nair, Pulkit Gupta, Mala Shanmugam

**Affiliations:** Winship Cancer Institute, Emory University, Atlanta, GA, United States

**Keywords:** multiple myeloma, mitochondria, metabolism, therapy, B cell

## Abstract

Multiple myeloma (MM) is a plasma cell dyscrasia characterized by the clonal proliferation of antibody producing plasma cells. Despite the use of next generation proteasome inhibitors (PI), immunomodulatory agents (IMiDs) and immunotherapy, the development of therapy refractory disease is common, with approximately 20% of MM patients succumbing to aggressive treatment-refractory disease within 2 years of diagnosis. A large emphasis is placed on understanding inter/intra-tumoral genetic, epigenetic and transcriptomic changes contributing to relapsed/refractory disease, however, the contribution of cellular metabolism and intrinsic/extrinsic metabolites to therapy sensitivity and resistance mechanisms is less well understood. Cancer cells depend on specific metabolites for bioenergetics, duplication of biomass and redox homeostasis for growth, proliferation, and survival. Cancer therapy, importantly, largely relies on targeting cellular growth, proliferation, and survival. Thus, understanding the metabolic changes intersecting with a drug’s mechanism of action can inform us of methods to elicit deeper responses and prevent acquired resistance. Knowledge of the Warburg effect and elevated aerobic glycolysis in cancer cells, including MM, has allowed us to capitalize on this phenomenon for diagnostics and prognostics. The demonstration that mitochondria play critical roles in cancer development, progression, and therapy sensitivity despite the inherent preference of cancer cells to engage aerobic glycolysis has re-invigorated deeper inquiry into how mitochondrial metabolism regulates tumor biology and therapy efficacy. Mitochondria are the sole source for coupled respiration mediated ATP synthesis and a key source for the anabolic synthesis of amino acids and reducing equivalents. Beyond their core metabolic activities, mitochondria facilitate apoptotic cell death, impact the activation of the cytosolic integrated response to stress, and through nuclear and cytosolic retrograde crosstalk maintain cell fitness and survival. Here, we hope to shed light on key mitochondrial functions that shape MM development and therapy sensitivity.

## Introduction

Multiple myeloma (MM) is a hematological malignancy estimated to account for 34,920 new cases and 12,640 deaths in 2022 in the United States (1). It is a genetically complex disease evolving from a premalignant asymptomatic state called monoclonal gammopathy of undetermined significance (MGUS) followed by smoldering MM (SMM) to full blown MM. The clinical manifestations of MM include hypercalcemia, renal insufficiency, anemia, and bone lesions. While current therapies largely target plasma cell features in combination with drugs targeting common drivers of cancer biology, patients eventually relapse and succumb to therapy refractory disease (2). Therefore, newer strategies are necessary to address intrinsic and extrinsic factors promoting MM progression and resistance.

Proteasome inhibitors (bortezomib (BTZ), carfilzomib, and ixazomib), immunomodulatory agents (thalidomide, lenalidomide, and pomalidomide), steroids, monoclonal antibodies (elotuzumab, targeting SLAMF7 and daratumumab and isatuximab targeting CD38) and ciltacabtagene autoleucel and ide-cel (BCMA-directed CAR-T immunotherapy) are currently some of the most important classes of anti-myeloma therapies (3–5). Induction treatment can be followed by autologous stem-cell transplantation (SCT) (or rarely, allogeneic SCT) with a form of maintenance therapy (e.g., lenalidomide or BTZ) to prevent relapse (6, 7). Patients who are not SCT-eligible instead receive chemotherapy treatment with lenalidomide/daratumumab in combination with BTZ (8, 9). Despite significant developments in MM therapy over the years, the majority of patients relapse with drug-resistant disease. Drug resistance in MM is a multifactorial phenomenon originating from both intrinsic and acquired resistance mechanisms stemming in part from genetic abnormalities, epigenetic gene regulation, evasion of apoptosis and metabolic alterations, underscoring the need for alternative methods to target MM (10, 11).

Cancer metabolism is now recognized as an enabling hallmark of cancer with direct or indirect implications on every hallmark of cancer. It is thus not surprising that over the last decade there are more studies showing the connections between metabolic state, disease progression and therapy resistance (12). Metabolic needs evolve throughout cancer progression. Cancer cells reprogram cellular metabolism to largely promote cell growth and proliferation, with specific dependencies dictated by the physiology of the cell of origin, identity of the transforming lesion and/or tissue of residence as well as in response to therapy. Apart from supporting bioenergetics and biosynthesis, intrinsic metabolic changes are instrumental in regulating protein kinase activity and signaling and gene and protein expression through epigenetic and post-translational modifications. Additionally, subpopulations within a tumor can exhibit metabolic heterogeneity driven by lack of proximity to oxygen, nutrient sources and other microenvironmental cues. Thus, elucidating context-specific metabolism is critical to understanding the contribution of metabolism to therapy efficacy and resistance mechanisms.

**Figure 1:**
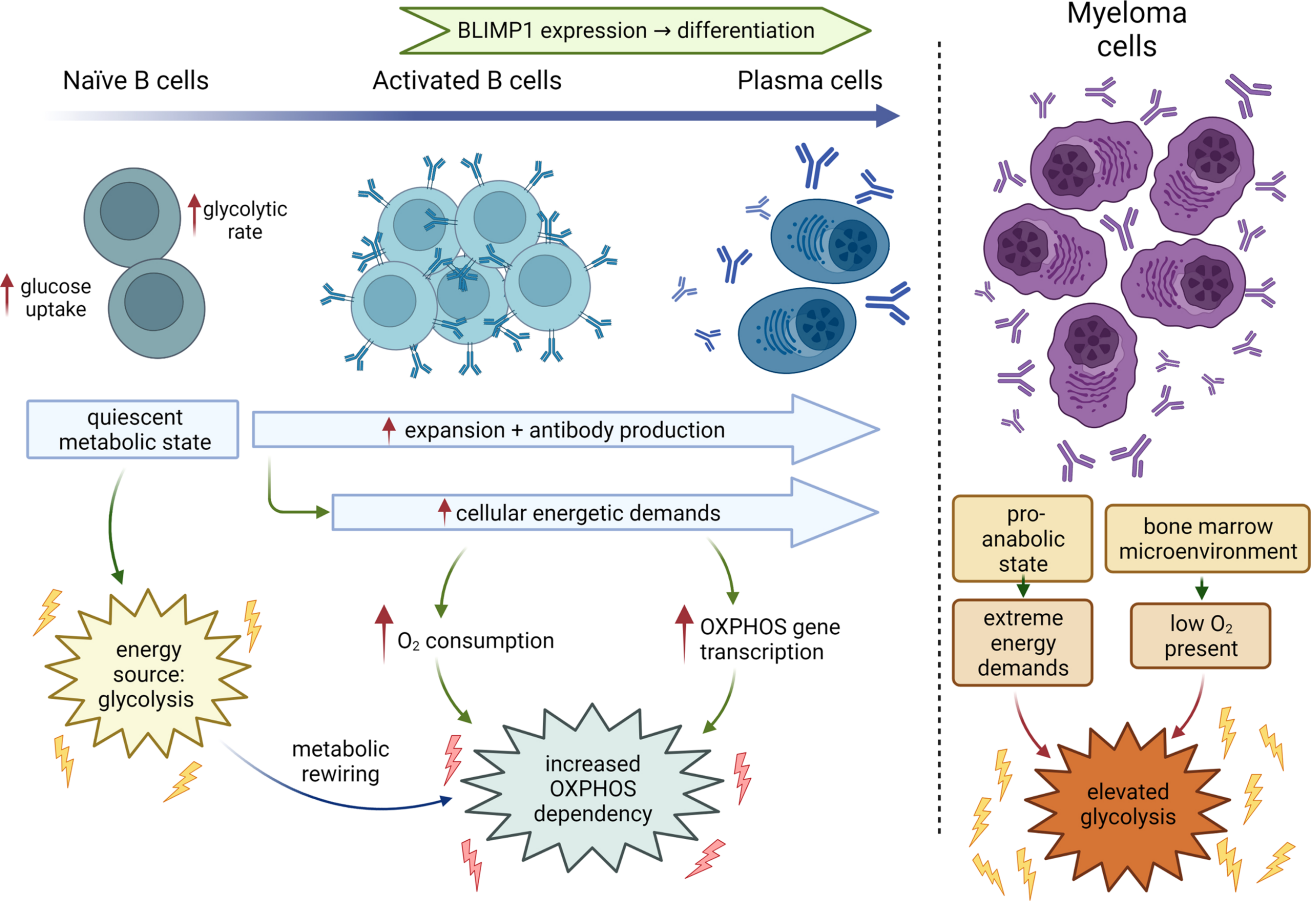
Metabolic changes during B-cell differentiation. Metabolic states of naïve B cells, activated B cells, fully differentiated plasma cells and MM cells. Normal B-cells transition from a primary dependency on glycolysis to additionally relying on elevated oxidative phosphorylation during development and differentiation. Myeloma cells continue to rely on glycolysis driven in part by the hypoxic conditions in the bone marrow, increased bioenergetic demands to sustain rapid growth and proliferation with OXPHOS likely supporting cellular functions like antibody production. BLIMP1, B lymphocyte-induced maturation protein 1.

MM is a malignancy of antibody producing plasma cells that develop from differentiating B cells. B cells exist in a resting or naïve state in the bone marrow, and transition into a rapidly proliferative state upon activation. These cells then undergo final maturation into terminally differentiated antibody producing plasma cells in secondary lymphoid organs such as the spleen and lymph nodes (13). Differentiation from B cells to plasma cells requires discrete changes in cellular metabolism to prepare for antibody production and expansion that is also likely shaped by the extrinsic availability of nutrients and oxygen. Naïve B cells generally exist in a quiescent stage characterized by low metabolic activity until they encounter antigen. During the transformation from the progenitor to precursor stage, B cells increase both glucose uptake and glycolytic rate, with large precursor B cells having the highest glucose consumption and sensitivity to glycolysis inhibition by 2-deoxy-D-glucose (2DG) (14, 15). B cells when activated undergo metabolic reprogramming in response to the changing energetic and biosynthetic demands required for plasma cell function. Additionally, activated B cells can also differentiate into antigen-experienced memory B cells that remain quiescent until they encounter antigen (**Figure 1**) (13).

B cells are stimulated either by T cell independent or dependent mechanisms, both of which are now known to create distinct metabolic dependencies (16). The T cell independent stimulation of B cells comprises stimulation with either lipopolysaccharide (LPS), a Toll-like receptor (TLR) 4 receptor agonist, or an anti-IgM antibody wherein the activated B cells rely on increased glucose import and display elevated oxygen consumption rates (OCR). Price *et al*, recently showed B cells stimulated with LPS displayed a progressive upregulation of tricarboxylic acid (TCA) cycle and electron transport chain (ETC) genes to support increased oxidative phosphorylation (OXPHOS) (17). Quiescent naïve B cells, activated B cells, and plasmablasts (PBs) show minimal, intermediate, and maximal dependance on OXPHOS, respectively (17). The surge in OXPHOS largely supports production of immunoglobulins and is dependent on the expression of the master regulator of PB differentiation, *BLIMP1*. As antibody secretion by PBs relies on OXPHOS, OXPHOS inhibition decreases the frequency of PBs. Overall, for maximal antibody production, B cells are inherently programmed to increase the transcription of OXPHOS pathway genes upon stimulation with mitogens like LPS during activation. Elevated OXPHOS is, thus, an important plasma cell-specific metabolic dependency, emphasizing the importance of mitochondrial function in maintaining plasma cell biology.

T cell-dependent B cell activation in secondary lymphoid organs leads to the migration of activated B cells into germinal centers (GCs), which are the sites of B cell differentiation. The hypoxic microenvironment in the GC would suggest that GC B cells rely more on glycolysis due to the lack of oxygen to support OXPHOS. However, although these GC activated B cells increase glucose uptake, they do not accumulate glycolytic metabolites. RNA sequencing (RNA-seq) data coupled with glucose isotopomer tracing reveals B cell activation to increase OXPHOS and TCA intermediates but not glycolysis. Elevated glucose import was found to primarily support ribonucleotide biosynthesis in contrast to fueling the TCA cycle (18). This study found that although the impact of limiting glucose on B cell activation, differentiation, or proliferation was not significant, perturbation of OXPHOS negatively affected B cell differentiation, reducing B cell size and preventing the upregulation of B cell activation markers. Activated B cells also show an increase in glutamine utilization that supports a myriad of B cell functions including fueling the TCA cycle and elevation of OXPHOS (18). Additionally, highly proliferative GC B cells use fatty acid oxidation to sustain OXPHOS while conducting minimal glycolysis (8, 19). Increase in OXPHOS during B cell activation is also accompanied by extensive mitochondrial remodeling. As compared to naïve B cells which have fewer, elongated mitochondria with multiple nucleoids, stimulated B cells undergo fission, increasing the number of rounded mitochondria without replicating their mt DNA (18).

T-cell dependent GC activation generates long-lived plasma cells that produce higher-affinity antibodies. These long-lived plasma cells migrate to the bone marrow where they reside to provide lifelong protection through the adaptive immune response. Short-lived plasma cells, on the other hand, generated in a primary immune response, develop in a T-cell-independent fashion and are mostly resident in secondary lymphoid organs.

Normal long-lived plasma cells are resident in a hypoxic bone marrow microenvironment and their metabolism is dictated by the state of activation. Transformed plasma cells, on the other hand, are in a perpetual pro-anabolic state to sustain biomass production for proliferation in the hypoxic bone marrow microenvironment, consequently exhibiting elevated glycolysis in addition to other metabolic alterations. It is the elevated glucose uptake and glycolysis that forms the basis for FDG-PET (fluorodeoxyglucose-positron emission tomography) based prognostic and diagnostic monitoring of MM tumor burden progression (20). However, in some instances low hexokinase expression precludes detection of MM, supporting the use of 11 (^11^C) methionine and development of [^18^F]4-fluoroglutamine imaging agents (21–23).

The Warburg effect describes the propensity of cancer cells to increase glucose uptake and exhibit aerobic glycolysis i.e. to ferment the end product of glycolysis, pyruvate, into lactic acid despite the presence of oxygen (24). Aerobic glycolysis has important metabolic consequences i.e.: 1) sustains elevated rates of ATP synthesis by increasing glucose uptake and glycolysis (25); 2) increases the pentose phosphate pathway (PPP) activity to generate NADPH for reductive biosynthesis and generation of nucleotide precursors, and lastly; 3) creates tumor microenvironment acidity promoting tumor growth and metastasis (26–28). Notably, hexokinase II (HKII), the first enzyme in the glycolytic pathway, is widely overexpressed in several cancers including MM. Consequently, inhibition of glycolysis by dichloroacetate (DCA) that inhibits pyruvate dehydrogenase kinase (PDHK) to activate pyruvate dehydrogenase (PDH) and OXPHOS, inhibits MM cell growth (29). A progressive upregulation of glucose metabolism-related genes are seen while comparing plasma cells from newly diagnosed (ND) MM patients *vs* normal donors and in relapsed *vs* newly diagnosed MM patients (30). The lactate generated by aerobic glycolysis also acts a fuel for neighboring cancer cells in the niche, providing an important carbon source for the TCA cycle and OXPHOS. For instance, in human non-small cell lung cancer (NSCLC), lactate’s contribution to the TCA cycle *in vivo* predominates that of glucose (31). In breast cancer, increased uptake of lactate was observed in regions of the tumor where oxygen is present (32). In MM, elevated lactate dehydrogenase (LDH, the enzyme that catalyzes the conversion of pyruvate to lactate) is a marker of poor prognosis at the time of diagnosis and increased LDH is associated with worse overall survival (OS), progression-free survival (PFS), aggressive disease, and higher tumor burden (33, 34). Additionally, extrinsic lactate accumulation inhibits the host immune response by increasing myeloid derived suppressor cells (MDSCs) and inhibiting NK cell function in tumor cells (35).

**Figure 2:**
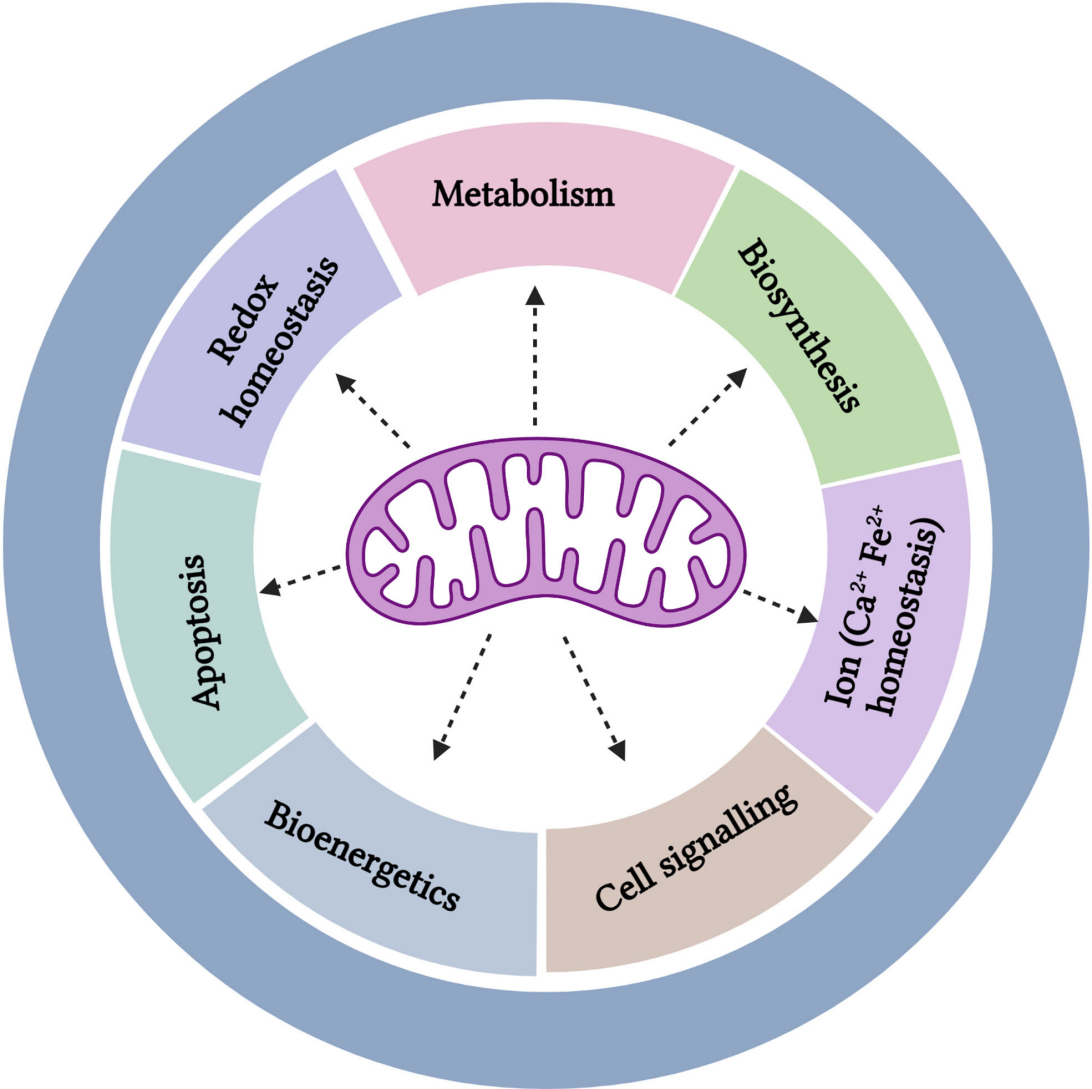
Multifaceted roles of the mitochondria.

Elevated glycolysis detected in cancer cells, that is also a property of normal proliferating cells, was originally described by Warburg to be a consequence of dysfunctional mitochondria. However, Warburg’s hypothesis on the reason for engaging aerobic glycolysis being consequent to mitochondrial dysfunction was first challenged in the early 1950s by Weinhouse et al, who showed that tumor cells effectively oxidized the fatty acid palmitate that would require functional mitochondria (36, 37). Henceforth, a growing number of studies have shown that cancer cells rely on specific aspects of mitochondrial function for growth and survival, despite exhibiting elevated aerobic glycolysis (36, 38). The seminal study testing the impact of removing functional mitochondria by suppression of the mitochondrial transcription factor A (TFAM) demonstrated a reduction of tumor burden, highlighting the requirement for mitochondrial metabolism and ROS in *KRAS*-driven tumor development (39). In the following sections we will discuss a subset of the diverse mitochondrial functions (**Figure 2**) and their implications for MM therapy.

## Mitochondria

Mitochondria, popularly known as the “powerhouse of the cell”, are ubiquitous intracellular organelles present in all eukaryotes. Mitochondria evolved from an ancestral organelle that originated upon the integration of an endosymbiotic bacterium with a host single cell prokaryote Archaea (40). Mitochondria were first discovered in 1857 by the Swiss physiologist Rudolf Albrecht von Kölliker who called them “sarcosomes”. Around 1890, Richard Altmann, a German pathologist and histologist proposed that they were intracellular parasites responsible for metabolic and genetic functions and termed them “bioblasts” (life germs). It was in 1898 that Carl Benda finally coined the term “mitochondrion” from the Greek words “mitos,” meaning “thread” and “khondros,” meaning “granule,” with the plural being “mitochondria.” In 1931, Otto Heinrich Warburg received the Nobel prize “*for his discovery of the nature and mode of action of the respiratory enzyme*”. Hans Krebs in 1937 discovered the TCA cycle for which he received the Noble prize in 1953 along with Fritz Albert Lipmann “*for his discovery of co-enzyme A and its importance for intermediary metabolism*.” Albert Claude, a Belgian-American biochemist, was the first to isolate mitochondria and demonstrate that they catalyzed respiration, for which he was awarded the Nobel Prize in 1945. Since then, mitochondria have been defined as the “*powerhouse of the cell*”. Electron microscopic studies by Palade and Sjostrand went on to identify mitochondria as double-membraned organelles in the1950s (41).

The next major milestone in mitochondrial biology emerged in the 1960s with the discovery that mitochondria contain their own DNA (mtDNA) distinct from the nuclear genome. In mammals, mtDNA inheritance is almost exclusively maternal, while paternal mtDNA is actively degraded in many species immediately after fertilization (42–45). Interestingly, quite recently Luo et al, demonstrated biparental inheritance of mtDNA in humans where paternal mtDNA was detected to be passed to the offspring (46). The mitochondrial genome encodes 13 proteins that contribute to forming subunits of all the electron transport chain (ETC) complexes except Complex II (which is completely nuclear-encoded); 22 tRNAs and 2 ribosomal proteins. The remaining mitochondrial proteins are encoded by the nuclear genome and are imported into the mitochondria (47).

**Figure 3:**
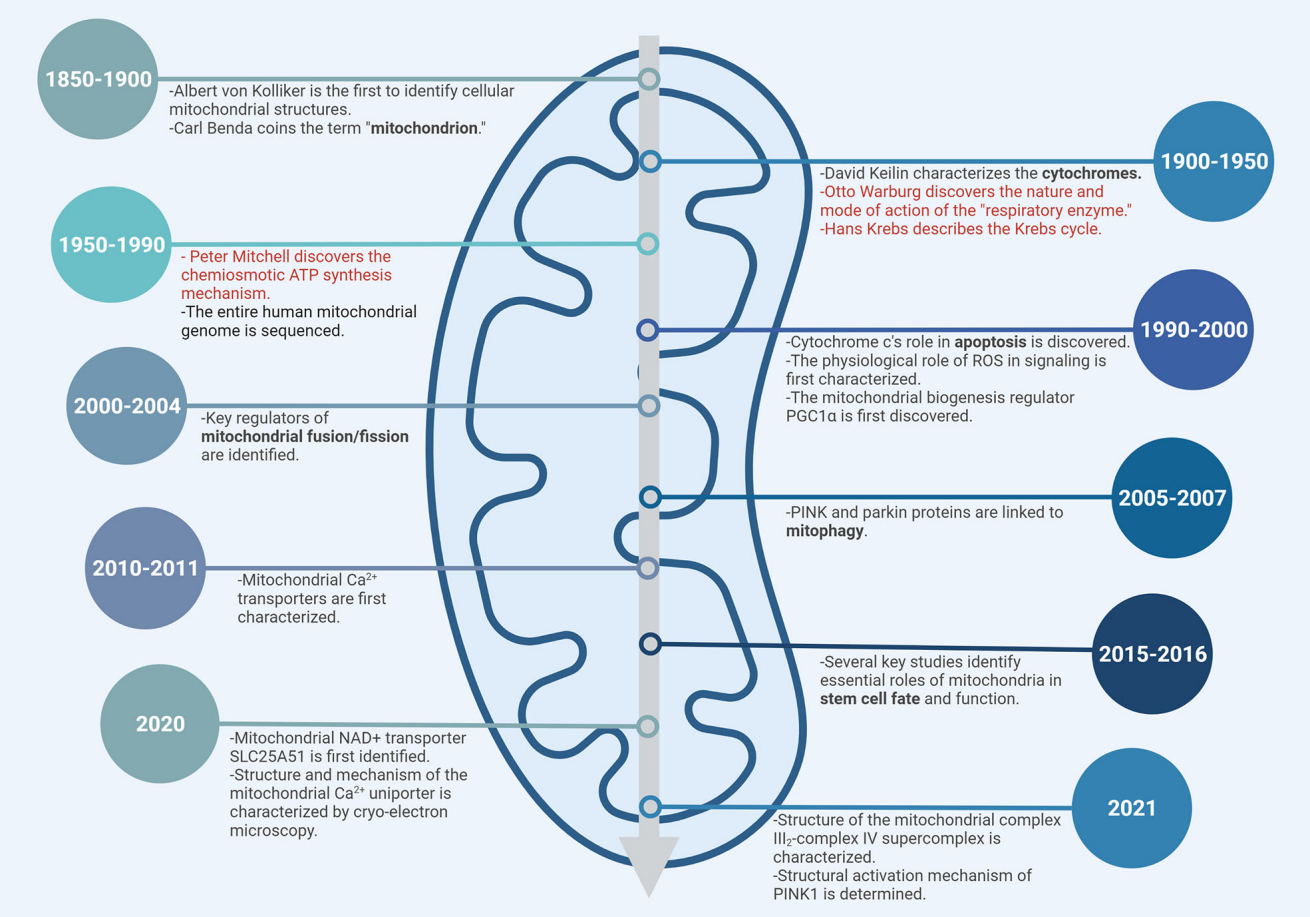
Timeline of mitochondrial biology. Subset of major milestones and discoveries are shown. Nobel Prize-winning discoveries are highlighted in red.

Despite the advancements in understanding the mitochondrial structure and its role in cellular respiration *via* OXPHOS, the question of how the oxidation of substrates was coupled to the synthesis of ATP from ADP and inorganic phosphate was finally solved quite some time later by Peter D. Mitchell. He proposed that the energy derived from oxidation of certain substrates (now known to be the electron donors NADH/FADH_2_) was used to pump protons from the mitochondrial matrix to the intermembrane space creating a “proton motive force” that was the driving agent for ATP synthesis. In 1978, Mitchell received the Nobel prize “*for his contribution to the understanding of biological energy transfer through the formulation of the chemiosmotic theory*” (48). A subset of major milestones and discoveries in mitochondrial biology is presented in **Figure 3**.

Research over the past few decades has implicated mitochondria and mitochondrial metabolism in regulating multiple essential cellular functions with implications for cancer development and therapy, which we review briefly below.

## Mitochondria: A central hub of cellular metabolism

The mitochondria are predominantly known for their role in efficient coupling of metabolite oxidation in the TCA cycle to ATP production *via* OXPHOS. OXPHOS comprises the transport of electrons through a series of five successive enzyme complexes located on the inner mitochondrial membrane, known as the electron transport chain (ETC). Electron donors, NADH for complex I and FADH2 or succinate for complex II of the ETC, are generated *via* the TCA cycle. These electrons are shunted between complex I and II to complexes III and IV by two mobile electron carriers, ubiquinone (ubiquinol, coenzyme Q10) and cytochrome c down an electrochemical gradient. Finally, the high proton gradient generated by complexes I, III, and IV across the inner membrane is released from the intermembrane space back into the mitochondrial matrix *via* complex V, a cylindrical turbine-like enzyme motor that couples the rotational energy generated from proton flow to drive the phosphorylation of ADP to ATP (49). The TCA cycle (Krebs cycle, citric acid cycle) is a sequence of eight biochemical reactions that occurs within the mitochondrial matrix. It begins with the reaction that combines acetyl CoA with oxaloacetate, catalyzed by citrate synthase, to generate citrate. Acetyl coA is typically generated as an end product of glycolysis i.e. pyruvate or fatty acids (via β-oxidation) or amino acids (via deamination). The TCA cycle generates various metabolic intermediates, reducing equivalents NADH and FADH and ATP by substrate phosphorylation. These metabolic intermediates can be used for the biosynthesis of various macromolecules needed for cell growth and proliferation, such as purines, pyrimidines, amino acids, fatty acids, etc. Abundance of different TCA cycle metabolites impacts cellular physiology and disease, as has been elaborately described in a recent review by Reyes et al. (50).

In addition to glucose, another major carbon source utilized for anapleurotic replenishment of TCA cycle intermediates is glutamine. Glutamine derived intermediates form precursors used in the synthesis of purine and pyrimidine nucleotides, non-essential amino acids (like alanine, aspartate, serine and proline) and the reducing equivalents fueling OXPHOS (51). Glutamine is utilized as a source of carbon and nitrogen and is consumed in rapidly dividing cells such as lymphocytes, enterocytes of the small intestine, and by cancer cells for both energy production and biomass generation (52, 53). MM cells are highly dependent on glutamine metabolism for survival (54, 55). An *in vivo* assessment revealed relatively higher glutamine anaplerosis into the TCA cycle in malignant BM plasma cells as compared to pre-malignant plasma cells (56). MM cells in the bone marrow exhibit elevated glutamine uptake fueling OXPHOS while the increased glucose uptake sustains elevated lactate production, in contrast to normal plasma cells in the BM (57). Notably, depletion of glutamine by MM cells in the BM impairs osteoblast differentiation along with hindering mesenchymal asparagine synthesis (58). Moreover, inhibition of glycolysis in MM cells promotes increased reliance on OXPHOS and glutamine metabolism, highlighting the importance of glutaminolysis in fueling OXPHOS under such conditions (55). Expression levels of the glutamine transporters ASCT2 (*SLC1A5*), LAT1 (*SLC7A5*) and SNAT1 are upregulated in CD138+ MM, compared to normal plasma cells (59). However, only inhibition of the ASCT2 transporter decreases glutamine uptake and myeloma cell growth significantly (59). Glutaminase 1 (GLS1) hydrolyzes glutamine into glutamate and fuels rapid proliferation of cancer cells. Pharmacological or genetic loss of GLS1 activity depletes TCA metabolites and retards cancer cell proliferation (60). Clinical data analysis revealed that overexpression of GLS1 in cancers including MM corelated with increased patient mortality (61, 62). Elevated glutamine import, glutamate production and export bring in cystine for glutathione (GSH) synthesis, an important regulator of redox homeostasis (63). Muir et al, demonstrated that cystine levels in extrinsic media and xCT/SLC7A11 (cystine transporter) expression are critical determinants of glutamine anaplerosis and glutaminase dependence in cancer cells and may explain the lack of efficacy of GLS1 targeting drugs in MM (60).

Demand for glutamine can also rise in hypoxic conditions in proliferating cells where glutamine derived αKG allows for citrate synthesis through reductive carboxylation that in turn supports fatty acid biosynthesis. Genetic impairment of the ETC also promotes IDH2 catalyzed reductive carboxylation of αKG. Additionally in some tumor cells with defective mitochondria, reductive carboxylation is required to support lipid synthesis and growth (64).

Glutamate also supports the biosynthesis of other amino acids such as alanine, aspartate, proline and serine, which are then used for arginine, asparagine, cysteine and glycine synthesis. Inhibition of glutamate dehydrogenase 1 (GLUD1), an enzyme localized in the mitochondrial matrix that is responsible for the conversion of glutamate to αKG and ammonia, leads to the attenuation of cancer cell proliferation and tumor growth by promoting imbalances in redox homeostasis (65). Interestingly, Spinelli et al. showed that GLUD1 can operate reversibly in breast cancer cells by assimilating ammonia to utilize it efficiently as a nitrogen source to generate amino acids (66). Overall, alterations in glutamine metabolism can be considered as a one of the major metabolic dependencies in cancer cells that promote tumor growth and development.

Other amino acids have also been recognized to contribute to oncogenesis. Serine and glycine both play essential roles in cancer cell growth by providing essential precursors for the synthesis of proteins, nucleic acids, lipids and antioxidant defense (67). Recently, Xia et al., demonstrated that extrinsic glycine deprivation inhibits both *in vivo* and *in vitro* MM cell proliferation by disrupting endogenous glutathione synthesis (68). Serine synthesis is upregulated in several cancers (69). Serine synthesis requires NAD+ in the cytosol, so coupling this pathway to mitochondrial electron transport relies on redox shuttles like the malate–aspartate shuttle to transport electrons across the mitochondrial membrane. Additionally, serine-glycine one-carbon metabolism (SGOC) is important to sustain the high proliferative rate of certain tumor cells (69, 70). Ceramides, which are serine-derived lipids, are also essential for mitochondrial function and cell proliferation (71). Aspartate is a proteinogenic amino acid that contributes to purine and pyrimidine nucleotide synthesis and TCA cycle anaplerosis (72). Recently, Bermudez et al, demonstrated that maintaining aspartate import in tumors enables cancer cell proliferation under ETC inhibition and hypoxia. Indeed, cancer cell lines sensitive to ETC inhibition are found to have reduced aspartate levels (72). Aspartate is synthesized from glutamate-derived nitrogen and oxaloacetate by the mitochondrial glutamic-oxaloacetic transaminase 2 (GOT2). Aspartate is also a key component of the malate-aspartate shuttle facilitating electron transfer between the mitochondrial matrix and intermembrane space to regenerate NADH inside the mitochondrial matrix. Birsoy et al. showed that the loss of GOT1, the cytosolic aspartate aminotransferase that normally consumes aspartate to transfer electrons into mitochondria, kills Jurkat, myeloma and lymphoma cells upon ETC inhibition. They showed that upon ETC inhibition, GOT1 reverses flux to generate aspartate in the cytosol, which partially compensates for the loss of mitochondrial aspartate synthesis (73). Both the Birsoy and Sullivan groups discovered that an essential role of cellular respiration is to provide electron acceptors for aspartate biosynthesis (73, 74). Arginine is involved in the biosynthesis of proteins, nucleotides, and metabolites such as nitric oxide, urea, ornithine and citrulline, making cancer cells dependent on its uptake (75). Several tumors have defects in the argininosuccinate synthase (ASS) enzyme required for synthesis of arginine *de novo* and are therefore completely reliant on extracellular arginine to support biological processes (75). Targeting arginine uptake has been tested in clinical trials as a therapeutic approach in some cancers (76). Arginine starvation has been shown to kill tumor cells causing mitochondrial dysfunction by downregulating OXPHOS and mitochondrial ETC gene expression (77). Metabolic profiles of MM patients at diagnosis exhibited higher levels of arginine along with some other amino acids when compared with healthy donors (78). MM cells are mostly arginine auxotrophic and thus can be selectively targeted by arginine starvation (79).

In sum, mitochondria play a critical role in the synthesis of non-essential and conditionally non-essential amino acids.

## Mitochondria are critical regulators of redox homeostasis

Mitochondria are the primary source of ROS (reactive oxygen species) in a cell. ROS is mainly generated at complex I and III of the respiratory chain. It comprises oxygen free radicals, such as the superoxide anion radical (O^2^·−) and hydroxyl radical (^·^OH), and nonradical oxidants, such as hydrogen peroxide (H_2_O_2_) and singlet oxygen (^1^O_2_). Notably, 1%–2% of oxygen consumed during physiological respiration is converted into superoxide radicals (80). ROS can have dual functions within cells and can be both essential and lethal. While mild increases in ROS can be conducive to normal cellular functioning by activating the phosphatidyl inositol 3 kinase (PI3K) and mitogen activated-protein kinase (MAPK)/extracellular-regulated kinase 1/2 (ERK1/2) growth signaling, excessive ROS can lead to cell stress and oxidative damage to important macromolecules, ultimately leading to cell death (81, 82). Programmed cell death can also result from ROS mediated peroxidation of cardiolipin that results in release of cytochrome c and caspase activation *via* the intrinsic apoptotic pathway (83). ROS can also activate hypoxia-mediated HIF-1α signaling leading to metabolic shift from OXPHOS to glycolysis by increasing expression of glycolytic enzymes (84). Typically, elevated ROS in the absence of redox homeostasis and sufficient antioxidants will lead to the rise of ROS beyond the “normal” or “physiological” threshold level resulting in “oxidative stress”.

Glutathione (GSH) and thioredoxin (TXN) are the two most powerful antioxidant systems in a cell. The GSH system comprises GSH, glutathione peroxidases (GPX) and glutathione s-transferases (GST) that reduce hydrogen peroxides or hydroperoxides using GSH as a substrate to reduce ROS. TXN, thioredoxin reductases (TXNRD) and NADPH function *via* reduction of the intracellular disulfides and direct quenching of ROS. Several cellular biochemical pathways, such as the pentose phosphate pathway, are important for regenerating NADPH from NADP+ to maintain the activity of these antioxidant mechanisms (85). Superoxide dismutases (SODs) also participate in ROS detoxification by converting superoxide anion (O2^−^) into oxygen and hydrogen peroxide, and enzymes like catalases convert hydrogen peroxide into oxygen and water.

Oxidative stress has been implicated in the development and pathophysiology of cancer (86). Excessive ROS production can damage key components of cells, including lipids, nucleic acids, and proteins and is implicated in increasing mtDNA mutations, ageing, and cell death. Increased metabolic rates, accumulation of genetic alterations and mutations in cancer cells are the primary triggers of ROS (87). As MM cells sustain massive Ig synthesis, assembly, and secretion, they undergo substantial endoplasmic and oxidative stress. In fact, redox state in MM cells regulates BTZ and melphalan cytotoxicity (88, 89). Notably, Bustany et al, demonstrated that the impaired redox status of cyclin D1-overexpressing MM cells mediates drug sensitivity to proteasome inhibitors (90). Overall, manipulating redox homeostasis in MM that already exhibits a heightened dependence on redox homeostasis consequent to antibody production could prove effective in enhancing therapy efficacy.

## Mitochondria in calcium and iron homeostasis

Mitochondria play an important role in calcium homeostasis by regulating calcium influx and efflux pathways (91–93). Ca^2+^ influx into the mitochondrial intermembrane space occurs through voltage-dependent anion channels, and influx into the mitochondrial matrix is carried out by mitochondrial channel uniporters (MCU) in high electronegative potential conditions (93–96). Efflux of Ca^2+^ in the mitochondria occurs either through H^+^/Ca^2+^ exchangers (HCX) or Na^+^/Ca^2+^ exchangers (NCLX), and through the balance of Ca^2+^ transport into and out of the mitochondria, Ca^2+^ homeostasis is achieved (97, 98). Physiologically, mitochondrial Ca^2+^ is a critical regulator of the TCA cycle through its activation of the three mitochondrial dehydrogenases – pyruvate dehydrogenase (PDH), isocitrate dehydrogenase (IDH) and alpha-ketoglutarate dehydrogenase (α-KGDH) resulting in an increase in supply of NADH to the ETC (99). Hence, given that OXPHOS is driven forward by increased mitochondrial Ca^2+^, calcium is also recognized to be an important indirect regulator of ROS production, which at subtoxic levels, is necessary for cell signaling (100, 101). Excess ROS generated by high levels of calcium has been found to contribute to cancer cell survival in many cancers by inducing the accumulation of mutations and allowing for greater proliferation and metastasis (102). With reference to calcium influx, silencing of MCU has been shown to lower cell motility, invasion, and migration in hepatocellular carcinoma, Hs578 breast cancer, HeLa, and triple-negative breast cancer cells (103–105). In MM, use of the MCU inhibitor ruthenium red reversed sensitivity to BTZ in U266, MM1S, and H929 cells, suggesting BTZ-induced mitochondrial calcium influx is necessary for BTZ cytotoxicity in MM (97, 106, 107).

Mitochondria are also the central hub for iron metabolism and homeostasis. Iron is necessary for performing electron transfer in a multitude of cellular biochemical reactions, which likely explains why many cancers exhibit a greater-than-normal cytosolic iron pool. For MM in particular, the cellular iron exporter ferroportin 1 (FPN1) is under expressed, leading to an increase in labile iron that contributes to cell growth and drug resistance (108). This increased labile iron pool leads to mitochondrial ROS accumulation and resultant lipid peroxidation, causing ferroptosis. Mitochondrial iron transporters mitoferrin 1 (SLC25A37) and mitoferrin 2 (SLC25A28)-mediated iron accumulation increase ROS mediated-carcinogenesis in osteosarcoma (109). Moreover, ROS generated by mitochondria can interact with cellular iron if iron levels are high, leading to increased oxidative DNA damage and subsequent accumulation of mutations. Modesti et al. have shown that iron levels influence mitochondrial biogenesis. Chelating iron with deferoxamine in MM cells led to a decrease in mitochondrial biogenesis-related gene expression, while overexpressing PGC1A, a coactivator of mitochondrial biogenesis, led to a decrease in *FPN1* expression and an increase in the iron importer, transferrin (*TFRC*). Additionally, a mitochondrial score (based on mitochondrial biogenesis gene expression and representative of mitochondrial metabolism) was shown to be positively correlated with *TFRC* expression and negatively correlated with *FPN1* expression in primary MM samples. MM patients with a high mitochondrial score and low *FPN1* expression were found to have an inferior prognosis in event free survival (EFS) and overall survival (OS) (110). These results suggest the impact of mitochondrial overactivity and dysregulation in disrupting iron homeostasis in MM. Additionally, Campanella et al., observed that iron supplementation enhanced the susceptibility of MM cells to bortezomib by increasing protein oxidation and cell death (111). Hence, modulation of iron homeostasis could be a promising strategy worth exploring to improve the efficacy of MM therapies.

## Mitochondria and the Integrated Stress Response

The integrated stress response (ISR) refers to an evolutionarily conserved adaptive program that is activated to protect a cell from a diversity of stress signals (112). For example, the ISR can be activated by endoplasmic reticulum (ER) stress, hypoxia, nutrient deprivation, and oxidative stress. Mitochondrial stress/dysfunction lead to the activation of the mitochondrial UPR (unfolded protein response) that activates the cytosolic ISR to suppress global translation and overall cell survival and fitness. When the stress is severe or prolonged, the ISR signaling can shift to initiate apoptosis. Depending on the type of stress encountered, specific ISR kinases (PKR, HRI, GCN2, and PERK) are activated that phosphorylate the eukaryotic translation initiation factor 2 subunit alpha (eIF2α) (113). eIF2α phosphorylation simultaneously results in a global inhibition of protein synthesis and the increased translation of several key transcription factors involved in protective gene expression, such as activating transcription factor 4 (ATF4) and DNA damage-inducible transcript three protein (DDIT3), also known as CHOP (C/EBP homologous protein) (112, 114).

Owing to higher proliferative demands, cancer cells have higher basal ISR levels as compared to normal cells, thus any additional perturbations heighten the ISR bringing the cell closer to the apoptotic threshold (115). Using a genome wide CRISPRi screen in HEK 293T cells, Guo et al. demonstrated that mitochondrial stress induces ATF4, the master transcriptional regulator of the ISR in the cytosol, *via* the OMA1-DELE1-HRI axis (116). Mick et al., have recently reported that mitochondrial defects in the ETC can alter specific metabolites leading to activation of the ISR (117). For instance, in proliferating myoblasts, inhibition of ETC elevates the mitochondrial and cytosolic NADH/NAD+ ratios, impeding aspartate synthesis and ultimately depleting asparagine, which activates the ISR *via* the amino acid responsive eIF2α kinase GCN2.

The unfolded protein response is also connected to the ISR. Cells generally activate the UPR under physiological and pathological conditions to cope with the accumulation of unfolded or misfolded proteins in the ER, which if prolonged can culminate in cell death (118). The UPR is activated by three ER transmembrane stress sensors: PERK (also involved in the ISR), inositol-required enzyme 1 (IRE1), and activating transcription factor 6 (ATF6). Together, the ISR and the UPR are essential in re-establishing proteostasis or can alternatively initiate a cell death pathway if the stress is not resolved (119, 120). MM cells, owing to immunoglobulin synthesis, are subject to continual ER stress and are, therefore, highly dependent on the UPR. Excessive oxidative stress encountered by MM cells due to elevated oxidative protein folding and mitochondrial respiration can also potentiate the ER stress response making MM cells more sensitive to perturbations of the ISR. The sensitivity to bortezomib treatment is associated with higher expression of ATF4 in MM. ATF4 is therefore suggested as a predictive therapy response biomarker for bortezomib and dexamethasone combination treatment in MM (121). Moreover, a unique point mutation of *PSMB5* (gene encoding the proteasome β5 subunit) alleviates fatal ER stress, promoting bortezomib resistance in MM cells (122). In AML, inhibition of mitochondrial translation has been shown to overcome venetoclax resistance through activation of the ISR (123). We have shown that reduced ETC activity both predicts and promotes sensitivity to the venetoclax (124). These studies exemplify the translational applicability of further exploiting the ISR for MM therapy.

### Mitochondria - at the crossroads for survival and death

Beyond the multiple core metabolic functions sustained by mitochondria essential for cell survival, paradoxically, mitochondria also play essential roles in programmed cell death (125, 126). As mitochondria are the primary site for metabolic-apoptotic crosstalk, alterations in mitochondrial function impact both metabolism and intrinsic apoptosis. The intrinsic apoptosis pathway involves release of apoptosis effector proteins cytochrome c and second mitochondria-derived activator of caspase (SMAC) from the mitochondrial intermembrane space to the cytoplasm, which lead to caspase activation and subsequent cell death. Generally, intrinsic stresses such as elevated cytosolic Ca2+, oxidative stress, DNA damage, hypoxia and survival factor deprivation, are known to activate the intrinsic apoptotic pathway. The intrinsic pathway of apoptosis is regulated by the B-cell lymphoma 2 (Bcl-2) family of proteins, a collection of approximately 20 mitochondrial pro- and anti-apoptotic proteins (127). Several of the BCL-2 family act as pro-apoptotic ‘activators’ and contain a single BH3 protein domain, namely BCL-2 interacting mediator of cell death (BIM), p53-upregulated modulator of apoptosis (PUMA), and truncated BH3 interacting domain death agonist (tBID). When released from anti-apoptotic multi-domain BCL-2 family members (BCL-2, BCL-xL, myeloid cell leukemia 1 (MCL-1), BCL-w, and A1), the BH3-only pro-apoptotics are free to activate the pro-apoptotic effectors BCL-2 associated X protein (BAX) and BCL-2 antagonist/killer (BAK). Activation of BAX and BAK lead to the formation of pores in the outer membrane of mitochondria, inducing increased permeability of the mitochondrial membrane, thereby facilitating the release of cytochrome c (cyto c) and sequential activation of the caspase cascade. The anti-apoptotic proteins Bcl-2, Bcl-xL, and Mcl-1 neutralize the pro-apoptotic activity of BAX and BAK. While the intrinsic apoptotic pathway is the most directly regulated by mitochondria, other forms of cell death, including necrosis, necroptosis (programmed necrosis), ferroptosis, and autophagy, are also tightly regulated by the mitochondrion (128–130).

The apoptotic potential of a cell is also dictated by the crosstalk between various metabolic pathways and components of the apoptotic network (131–133). Interestingly, while cytochrome c has a fundamental role in initiating intrinsic apoptosis upon release into the cytosol, it separately functions to transfer electrons between complex III and complex IV of the respiratory chain. In this context, glucose metabolism inhibits apoptosis by caspase inactivation through NADPH-mediated redox inactivation of cytochrome c (134). Additionally, while ceramides promote apoptosis by BAX dependent mechanisms, sphingolipid metabolism co-operates with BAK/BAX dependent mechanisms to promote permeabilization of the mitochondrial outer membrane (MOMP) (135, 136). While phosphorylation of proapoptotic NOXA and BAD promotes glucose consumption through PPP and glycolysis, respectively, the anti-apoptotic BCL-XL increases the efficiency of ATP synthesis by decreasing a proton leak within ATP synthase (137, 138). Lin et al, recently charted the first apoptotic map of metabolism and showed reduction of heme biosynthesis to potentiate apoptosis through the loss of ETC activity (139).

**Figure 4:**
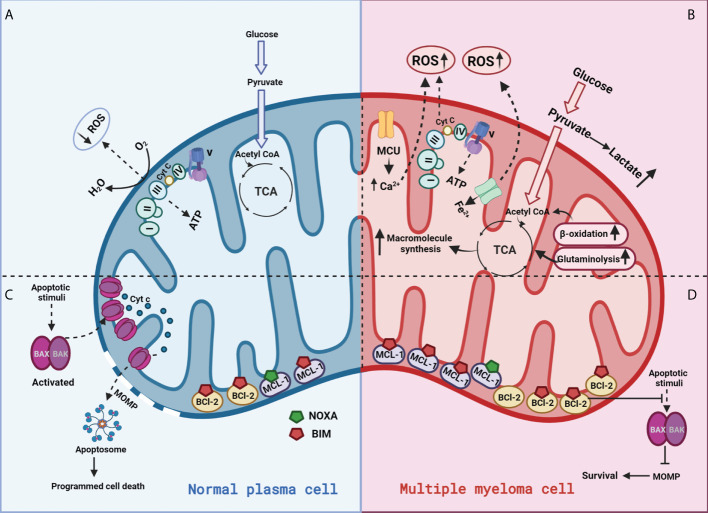
Mitochondrial functions in a normal plasma vs multiple myeloma cell:**(A)** In normal plasma cells, glucose is primarily metabolized through glycolysis into pyruvate and acetyl-CoA. Acetyl-CoA enters the mitochondrial TCA cycle to produce a series of intermediates and reducing equivalents that are utilized by the ETC to generate ATP through OXPHOS; **(B)** Cancer cells generate energy preferentially by glycolysis even in the presence of oxygen, a phenomenon known as the “Warburg effect.” This metabolic switch towards “aerobic glycolysis” increases glucose uptake mediated via glucose transporter (GLUT) upregulation and oxidation of pyruvate to lactate. **(C)** Normal cells appropriately respond to stress by activating apoptosis; **(D)** Cancer cells overexpress anti-apoptotic proteins (BCL-2) to counteract pro-apoptotic BAX/BAK activation, allowing for the evasion of programmed cell death.

Evasion of apoptosis is a hallmark of cancer. The therapeutic potential of targeting anti-apoptotics like BCL2 and MCL1 in hematologic malignancies is extensively reviewed in (140) and we have reported on the intersection of cellular metabolism and BCL-2 proteins in (127). More specifically, we have shown that glucose deprivation or targeting GLUT1 in MM can reduce MCL-1 expression, while glutamine deprivation increases BCL-2 dependance by increasing BIM bound to BCL-2 (141, 142). Additionally, we found reduced succinate ubiquinone reductase activity to predict and promote BCL-2 dependence (124). Resistance to apoptosis, primarily by overexpression of anti-apoptotic proteins and/or altered sequestration of pro-apoptotics plays a crucial role in both pathogenesis and resistance to treatment of MM (**Figure 4**) (143, 144). Thus, targeting metabolism to antagonize anti-apoptotic proteins or induce pro-apoptotics expression or release from the anti-apoptotics can bring a cell closer to the apoptotic threshold and sensitize MM cells to specific therapeutic agents. Differences in mitochondrial functions in normal cells vs plasma cells are represented in **Figure 4**.

## Genetic lesions and MM metabolism

Common genetic lesions in MM include mutually exclusive mutations in KRAS or NRAS and BRAF, deletions in p53 and amplifications in MYC (145, 146). These genetic lesions can have profound effects on cancer and MM metabolism. For instance, genetic alterations of the tumor suppressor p53 reduce oxygen use and lower the level of mitochondrial respiration, thereby promoting the Warburg effect (147). Precisely, p53 has been shown to regulate glycolysis through repression of the glucose transporters GLUT1 and GLUT4 and upregulation of the gene *TIGAR*, which encodes a protein that degrades fructose-2,6-bisphosphate to block glycolysis (via the conversion of fructose-1,6-bisphosphate to fructose-6-phosphate) (148, 149). Furthermore, p53 upregulates SCO2, a stabilizer of the cytochrome *c* oxidase 17 subunit that functions in OXPHOS. Mutations in isocitrate dehydrogenase, succinate dehydrogenase and fumarate hydratase lead to the accumulation of the neomorphic oncometabolite 2-hydroxyglutarate (2-HG), succinate or fumarate respectively, contributing to the development and the progression of several cancers (150).

KRAS plays an important role in driving glycolysis in pancreatic cancer, leading to progression of the disease by increasing GLUT1, hexokinase II (HK II), and phosphofructokinase 1 (PFK1) expression (151–154). Mutant KRAS has been shown to rewire amino acid metabolism in B-acute lymphoblastic leukemia (B-ALL) through AKT/mTOR signaling, specifically promoting the catabolism of Met and Arg in favor of proline and polyamine production (155). Although further research is needed to elucidate the metabolic effects of p53 and KRAS/NRAS in MM, there are several other genetic lesions that have been studied in MM that can likely regulate mitochondrial metabolism (16).

MYC is one such genetic lesion that regulates several metabolic nodes like glycolysis, glutaminolysis, nucleotide synthesis, lipid synthesis, and mitochondrion/ribosome biogenesis (156). MYC deregulation is one of the prominent features associated with MM disease progression that occurs in 67% of MM cases (157, 158). Overexpression of MYC is associated with glutamine addiction through an increase in glutamine transporter (ASCT2) and glutaminase (GLS) transcription, which is relevant in MM due to an increased dependency on glutamine as a metabolic nutrient (157, 159, 160). Degradation of MYC by the proteasome *via* fibroblast growth factor trapping was specifically shown to lead to glutathione depletion in MM cells, resulting in increased mitochondrial oxidative stress (161).

Another genetic aberration is the mutation in the receptor tyrosine kinase FGFR3, which when overexpressed regulates MM lipid metabolism. Specifically, two ligands of FGFR3, FGF2 and FGF8, have been shown to enhance FGFR3 downstream signaling in several MM cell lines, increasing phospholipase C gamma (PLCγ) phosphorylation (162). Overexpression of MMSET (multiple myeloma SET domain) is a characteristic of all t(4;14) MM patients. MMSET I (multiple myeloma SET domain-containing protein I), an isoform of the MMSET protein was shown to transcriptionally upregulate glyoxalase I (GLOI), which is involved in detoxifying aldehyde products during glycolysis (163–165). Notably, knockdown of MMSET I in the KMS11 MM cell line reduced fructose-1,6-bisphosphate, glyceraldehyde-3-phosphate, lactic acid, citric acid, and NADH levels, and the glycolytic metabolites were restored upon GLO-1 re-expression (166).

### Implications of mitochondrial metabolism on MM therapy

**Figure 5:**
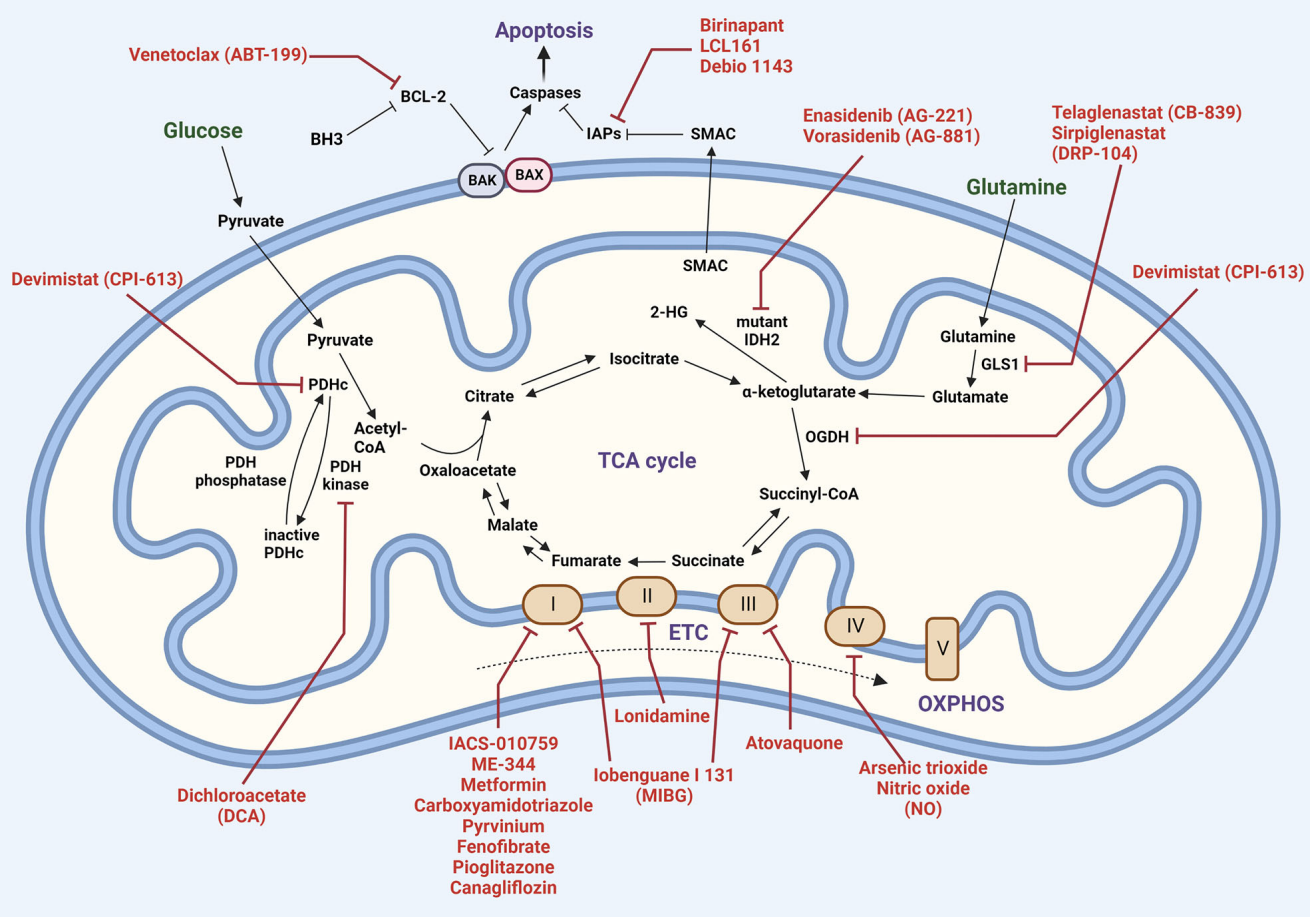
Mitochondrial therapeutic targets. Carbon metabolism (Krebs cycle and oxidative phosphorylation) and apoptosis pathways are shown, with inhibitors highlighted in red. Abbreviations: 2-hydroxyglutarate (2-HG); BCL-2 homologous antagonist killer (BAK); BCL-2-associated X protein (BAX); B-cell lymphoma 2 (BCL-2); BCL-2 homology domain 3 (BH3); glutaminase1 (GLS1); inhibitor of apoptosis (IAP); isocitrate dehydrogenase 2 (IDH2); alpha-ketoglutarate dehydrogenase (OGDH); pyruvate dehydrogenase complex (PDHc); second mitochondria-derived activator of caspases (SMAC).

Metabolic alterations have been linked to disease evolution and resistance to chemotherapeutic agents in MM (145, 167). A comprehensive list of mitochondrial metabolic inhibitors that are currently undergoing trials for different types of cancers is shown in **Table 1**. Ongoing efforts are directed towards identifying metabolic markers for diagnosis and risk prediction in MM (168–171). Common drugs that target the various metabolic functions of mitochondria are shown in **Figure 5**. Metabolomics of healthy controls vs patients with MGUS, NDMM or RRMM show significant alterations in amino acid, lipid and energy metabolism (172). Changes in bioenergetics, leading to enhanced mitochondrial biomass and function have been shown to contribute to drug resistance in hematological malignancies including MM (173). Additionally, patients with relapsed and refractory MM have increased mitochondrial biogenesis signatures that correlate with MM progression (110). We have previously demonstrated the therapeutic ability of targeting metabolic vulnerabilities in MM including development of glucose transport (GLUT4) inhibitors and the advantages of combinatorial treatments targeting glucose and glutamine metabolism in MM (51, 141, 142, 174, 175). We also reported on the utility of targeting increased mitochondrial OXPHOS in the subset of MM cells that survive glucose deprivation or GLUT4 inhibition with ritonavir (141). These findings, in sum, exemplify the critical influence of mitochondria in overcoming drug resistance in MM.

**Table 1:** List of mitochondrial metabolic inhibitors undergoing trial for cancer therapy.

Drug	Mode of Action	cancer Type(s)	clinical Trial Status
Telaglenastat (CB-839)	GLS inhibition	prostate cancer, solid tumors, renalcell carcinoma,triple negative breast cancer, NSCLC, melanoma	Phase 2
Sirpiglenastat (DRP-104)	glutamine antagonist	advanced solid tumors	Phase 2
Enasidenib (AG-221)	mutant IDH2 inhibition	AML	FDA-approved
Vorasidenib (AG-881)	mutant IDH1and mutant IDH2 inhibition	residual/recurrent grade II glioma	Phase 3
IACS-010759	mitochondrialcomplex I inhibition	advanced cancers, AML	Phase 1
ME-344	mitochondrial complex I inhibition	solid tumors	Phase 2
Metformin	mitochondrial complex I inhibition, AMPK activation, cAMP inhibition	breast cancer,endometrial, cancer, prostate cancer, medulloblastoma	Phase 3
Carboxyamidotriazole	mitochondrial complex I, inhibition, calcium channel, blockage	NSCLC	Phase 3
Pyrvinium	mitochondrial complex I, inhibition, CK1α activation	pancreatic cancer	Phase 1
Fenofibrate	mitochondrial complex I inhibition,PPARα activation	neuroblastoma, leukemia, lymphoma,sarcoma, pediatric CNS tumor, advanced NSCLC, recurrent medulloblastoma, recurrent ependymoma, recurrent ATRT	Phase 2
Pioglitazone	mitochondrialcomplex I inhibition,PPARy activation	advanced melanoma, CML, liposarcoma, multiple, myeloma, pancreatic cancer, thyroid cancer, lung cancer	Phase 2
Canagliflozin	mitochondrial complex I inhibition, SGLT2 inhibition	breast cancer, advanced solid tumors	Phase 2
lobenguane 1131(MIBG)	mitochondrialcomplex I/III inhibition, adrenergic neurotransmitter analogue	pheochromocytoma, paraganglioma	FDA-approved
Lonidamine	mitochondrial complex II inhibition	melanoma,colon cancer, liver cancer, cervical cancer	Preclinical
Atovaquone	mitochondrial complex Ill inhibition	NSCLC	Phase 1
Arsenic trioxide	mitochondrial complex IV inhibition	acute promyelotic leukemia	FDA-approved
NO	mitochondrialcomplex IV inhibition, metal nitrosyl complex cancer formation	solid tumors, colorectal	Phase 1
Devimistat (CPI-613)	mitochondrial OGDH and PDH inhibition	relapsed/refractory AML, metastatic pancreatic, cancer	Phase 3

NSCLC-non-small cell lung cancer, IDH-isocitrate dehydrogenase, AML-Acute myeloid leukemia, AMPK-AMP-activated protein kinase, CK1α-casein kinase 1α, PPAR-Peroxisome proliferator-activated receptor, SGLT2- Sodium-Glucose Transport Protein, OGDH- 2-oxoglutarate dehydrogenase, PDH-Pyruvate dehydrogenase.

Proteasome inhibition induces ER stress, a terminal UPR, and oxidative stress, leading to apoptosis (176). Various studies have shown how mitochondrial metabolism impacts BTZ sensitivity. The contribution of mitochondrial OXPHOS to proteosome inhibitor sensitivity is not entirely clear. BTZ resistant MM cells display proteomic changes in redox and energy metabolism (177). Increased OXPHOS has shown to promote protein folding, mediating BTZ resistance (178). However, hypoxic conditions that favor higher glycolytic activity reduce BTZ sensitivity in MM (30). Inhibition of aerobic glycolysis with dichloroacetate (DCA), glutamine transport by targeting ASCT2 or glutaminase with CB-839 increases sensitivity to bortezomib in MM (29, 179, 180). More recently, higher activity of both the pentose phosphate pathway and serine synthesis increased BTZ resistance by increasing antioxidant synthesis. Consistent with this, serine starvation enhanced the cytotoxicity of BTZ (11). In a functional screen to identify targets that synergize with PIs targeting the mitochondrial enzyme, IDH2 emerged as a top candidate that displayed synthetic lethal activity with the PI carfilzomib (CFZ) (181). Mutant IDH2 inhibitors have been shown to sensitize cells to BTZ in MM (181). Incidentally, glutamine derived 2-HG is associated with disease progression in MM (182). BTZ resistance correlates with high levels of the mitochondrial electron carrier CoQ, and targeting the mevalonate pathway with simvastatin decreases CoQ levels, overcoming BTZ-resistance in MM cells (183). The glutaminase inhibitor, telaglenastat (CB-839), has been tested in combination with carfilzomib and dexamethasone in relapsed and/or refractory MM, and newer inhibitors like DRP-104 (sirpiglenastat) are in solid tumor clinical trials (184–186). Additionally, amino acid depletion by ʟ-asparaginase that induces serum amino acid depletion by L-Asn and L-Gln hydrolysis sensitizes MM cells to carfilzomib by inducing mitochondria ROS-mediated cell death (187).

Evasion of apoptosis, another hallmark of malignant cells, constitutes an important mechanism associated with resistance. Thus, targeting the anti-apoptotic members of the BCL-2 family of proteins (BCL-2, BCL-X_L_, and MCL-1) is an attractive target for therapy owing to their crucial role in determining cellular fate. Moreover, BCL-2, MCL-2, BCL-xL, PUMA, NOXA, and BIM expression and/or their interactions have been shown to be regulated by metabolites such as glucose, glutamine, and (R)-2HG (124). Hence, metabolic changes can alter BH3 mimetic dependence and sensitivity, thereby impacting resistance to therapy. We have previously demonstrated glutamine deprivation to sensitize MM cells to venetoclax by enhancing BIM binding to MCL-1 (142). Sensitivity to venetoclax targeting BCL2 is associated in part with increased BCL-2 expression in MM and elevated pro-apoptotics binding to BCL-2 (188). However, venetoclax is not successful as a single agent in MM except for in a minority contingent of t(11;14) cells. We have shown that *single agent venetoclax sensitive* MM cells irrespective of their t(11;14) translocation status exhibit low oxygen consumption and ETC (complex I and complex II succinate dehydrogenase and succinate ubiquitinase) activity (124). ETC activity measurements can thus be further developed for predicting venetoclax sensitivity. Additionally, we showed suppression of the ETC (genetically or pharmacologically) is sufficient to promote sensitivity to Ven (124). Intriguingly, we recently determined that ETC suppression promotes resistance to PIs and this resistance can be targeted by co-treatment with erastin or venetoclax (189). Therefore, targeting the BCL-2 family proteins in combination with targeting metabolic vulnerabilities is a viable strategy for eliciting deeper responses and overcoming therapy resistance in MM (124).

Alterations in several metabolic pathways are also known to impact resistance to the alkylating agent melphalan. Melphalan-resistant cells display an upregulation in glycolytic and pentose phosphate pathway enzymes and a downregulation in TCA cycle and ETC pathway enzymes (30). Recent transcriptomic evaluation of melphalan resistance revealed increased expression of genes involved in amino acid and glutathione (GSH) metabolism and also alterations in purine and pyrimidine metabolism (190). Moreover, cells that are resistant to melphalan display markedly increased sensitivity towards inhibitors of both glycolysis and mitochondrial electron transport chain (191).

HIF-1α, c-MYC and p53 are transcription factors that are critical regulators of cellular metabolism (145). Pharmacologic inhibition of HIF-1α restores sensitivity to immunomodulatory drugs (IMiDs) and proteasome inhibitors in MM cell lines (192). Another study has shown LncRNA, PDIA3P interacts with c-Myc to increase glucose 6-phosphate dehydrogenase (G6PD) expression and PPP flux, and thereby promote the proliferation and drug resistance of MM (193). G6PD activity is also known to determine the cytotoxicity of RNA-directed nucleoside analogues in B cell malignancies (141, 194).

Apart from the mitochondrial changes within cells, mitochondrial exchange between cells can also play an important role in the development and progression of cancer. Horizontal mitochondrial transfer occurs *via* several widely studied mechanisms, including formation of gap junctions, production of tunneling nanotubes, or partial/complete cell fusion, among others (68-70). This was initially observed in human mesenchymal stem cells (MSCs), where MSCs transferred their mitochondria to cells with non-functional or deprived mitochondria *via* tunneling nanotubes (TNTs), exosomes or vesicles and rescued aerobic respiration (195). In immune cells, MSCs donate mitochondria to innate immune cells to improve their phagocytic activity (196). Mitochondrial exchange has been shown to be functionally important in restoring mitochondrial respiration in cancer cells with mitochondrial damage, as well as in aiding cells with evading apoptosis (197, 198). For instance, stromal cells have been shown to transfer healthy mitochondria to acute lymphoblastic leukemia (ALL) cells through tunneling nanotubes to provide support from oxidative stress (199). More recently, in AML the chemo-sensitizing effect of metformin was shown to be through reducing the mitochondrial transfer and mitochondrial oxidative phosphorylation in the recipient cells (200).

Likewise, mitochondrial exchange occurs between stromal and MM cells to move damaged mitochondria out of dying tumor cells and incorporate functional mitochondria into weaker MM cells (201, 202). MM cells rely on both oxidative phosphorylation and glycolysis following the acquisition of mitochondria. Therefore, the mechanisms by which mitochondrial transfer promotes drug resistance warrant extensive research and the inhibition of mitochondrial transfer could be a treatment option to prevent drug resistance.

Overall, targeting mitochondrial metabolism is a crucial yet comparatively underexplored strategy in cancer therapy.

## Conclusion

In conclusion, despite the incorporation of agents targeting intrinsic and extrinsic plasma cell biology and cancer biology, we continue to see the emergence of relapsed and refractory MM. Cellular metabolism is connected to all the hallmarks of cancer. The underlying metabolic reprogramming and rewiring in MM has a fundamental role in promoting MM growth, survival and drug resistance. Altered metabolism in MM has been directly or indirectly linked to mitochondrial function, structure, and dynamics. Therefore, further interrogation of how mitochondrial metabolism and function impacts broader cellular physiology will help reveal new therapeutic strategies to treat this largely fatal hematological malignancy.

## Author contributions

MS and RN conceptualized the manuscript. RN and PG made the figures with supervision from MS. RN, PG and MS wrote the manuscript. All authors contributed to the article and approved the submitted version.

## Funding

The work was supported by National Cancer Institute, National Institute of Health grant R01 CA247367-01A1 to MS and Leukemia Lymphoma Society TRP grant- ID 00112418 to MS.

## Acknowledgment

We would like to thank Dr Anthea Hammond for editorial review of the manuscript.

## Conflict of interest

The authors declare that the research was conducted in the absence of any commercial or financial relationships that could be construed as a potential conflict of interest.

## Publisher’s note

All claims expressed in this article are solely those of the authors and do not necessarily represent those of their affiliated organizations, or those of the publisher, the editors and the reviewers. Any product that may be evaluated in this article, or claim that may be made by its manufacturer, is not guaranteed or endorsed by the publisher.
